# Effects of Intended Scapular Posterior Tilt Motion on Trapezius Muscle Electromyography Activity

**DOI:** 10.3390/ijerph18179147

**Published:** 2021-08-30

**Authors:** Soo-Yong Kim, Il-Young Yu, Jae-Seop Oh, Min-Hyeok Kang

**Affiliations:** 1 Department of Physical Therapy, Pusan National University Yangsan Hospital, Yangsan 50612, Korea; gasigogi11@naver.com; 2 Department of Rehabilitation Center, Dang Dang Korean Medicine Hospital, Changwon 51495, Korea; dlfduddl@hanmail.net; 3 Department of Physical Therapy, INJE University, Gimhae 50834, Korea; ysrehab@inje.ac.kr; 4 Department of Physical Therapy, College of Health Sciences, Catholic University of Pusan, Busan 46252, Korea

**Keywords:** electromyography, lower trapezius, scapular posterior tilt, selective muscle activation

## Abstract

The intended scapular motion is a strategy to strengthen the lower trapezius (LT). However, few studies have explored the effects of the intended scapular posterior tilt motion on selective LT activation. Thus, the present study investigated the effect of the intended scapular posterior tilt on the electromyography (EMG) activity of trapezius muscles during prone shoulder horizontal abduction (PSHA). Eighteen asymptomatic men performed three types of PSHA: (1) preferred PSHA, (2) PSHA with the intended scapular posterior tilt, and (3) PSHA with the intended scapular posterior tilt and trunk extension. EMG activity of the upper trapezius (UT), middle trapezius (MT), and LT were measured during PSHAs. Scapular posterior tilt angle, with and without the intended scapular posterior tilt, were measured using inclinometer. The results indicated that LT muscle activity increased when scapular posterior tilt was applied with and without trunk extension (14–16%), compared to the preferred condition, during PSHA (*p* < 0.05). However, the addition of trunk extension to PSHA with the intended scapular posterior tilt increased the UT muscle activity (28%) and the UT/LT (29%) and UT/MT (31%) ratios (*p* < 0.05). The scapular posterior tilt angle was higher (15%) when applying the intended scapular posterior tilt (*p* = 0.020). These findings suggest that the intended scapular posterior tilt may be a useful strategy for selective LT muscle activation.

## 1. Introduction

Altered scapular movement or position (i.e., scapular dyskinesia) can lead to various shoulder injuries, including subacromial impingement, rotator cuff disease, and superior labral injury [1,2,3,4]. A crucial factor for altered scapular movement and/or shoulder injury is imbalanced scapular muscle activation [3,4,5,6], which includes reduced activity of the lower trapezius (LT) or middle trapezius (MT) muscle and enhanced activity of the upper trapezius (UT) [6,7,8]. In particular, altered scapular movement and imbalanced scapular muscle activation often occur in overhead athletes such as tennis players, volleyball players, and swimmers [1,7]. Previous studies have shown elevated UT/LT activity ratios during arm elevation [8] and enhanced UT muscle activity, together with reduced MT and LT muscle activities, during shoulder abduction and external rotation [7] in individuals with subacromial impingement, compared with healthy individuals. Functionally, the MT and LT contribute to scapular stability by limiting the unnecessary scapular vertical and horizontal displacement and by maintaining the correct scapular position [5,9]. The LT also contributes to scapular upward rotation and posterior tilt, increasing the space in the subacromial region; this allows efficient glenohumeral joint movement [5,10,11]. Conversely, weakness of the LT reduces the subacromial space, leading to subacromial impingement [12,13]. Therefore, to prevent shoulder injury and altered scapular movement, exercises have been developed to focus on LT muscle activation [3,10,14,15].

Prone shoulder horizontal abduction (PSHA) is a representative exercise for strengthening the LT. Previous studies comparing LT muscle activity among various exercises have suggested that PSHA in line with the LT muscle fiber can facilitate LT activation [16,17]. Similarly, LT muscle activity is greater at 125° shoulder abduction than at 160° during PSHA, because 125° shoulder abduction is closest to the direction of LT muscle fiber [15]. Other studies have suggested prone trunk extension to facilitate LT activation [18,19]. Furthermore, a study showed that participants had greater LT muscle activation during prone trunk extension when in the shoulder abduction position, compared to when their arms were placed at their sides [19].

Recent studies have reported the effects of exercise using the functional action of LT [10,14]. Intended performance of the functional action of a specific muscle is presumed to produce a higher electromyography (EMG) of that muscle during static and dynamic movement [3,20,21]. However, the intended scapular adduction and depression—functional actions of the LT—failed to increase LT muscle activation during PSHA [14]. In contrast, the intended scapular posterior tilt motion led to greater LT muscle activation in the static sitting position [10]. These conflicting findings may be the results of differences in scapular motions performed in these studies. Scapular adduction and depression are produced by the LT, as well as other scapular muscles (e.g., MT, rhomboid, levator scapulae, and latissimus dorsi) [11], whereas scapular posterior tilt is primarily caused by the LT and serratus anterior [1,22,23]. Therefore, selective activation of the LT may be interrupted by contraction of other scapular muscles during scapular adduction and depression.

Based on previous findings regarding the positive effects of scapular posterior tilt on LT muscle activity [10], the intended scapular posterior tilt could serve as an alternative action to facilitate LT activation. One previous study examined the influences of the intended scapular posterior tilt solely in the static sitting position [10]. To clarify the effects of the intended scapular posterior tilt on selective LT activation, there is a need to determine how this movement alters the EMG activity of trapezius muscles during dynamic LT strengthening exercises, such as PSHA and trunk extension. In addition, to guide clinicians on a shoulder injury prevention exercise program, it is necessary to demonstrate the effects of the intended scapular posterior tilt in asymptomatic subjects. Therefore, this study examined the effects of the intended scapular posterior tilt on the EMG activity of trapezius muscles during PSHA, with and without trunk extension in asymptomatic subjects.

## 2. Materials and Methods

### 2.1. Participants

Participants included 18 asymptomatic men (mean age = 23.39 ± 2.23 years; mean height = 175.44 ± 5.44 cm; mean body weight = 77.28 ± 8.87 kg) who had no shoulder pain and/or discomfort at the time of the experiment. Individuals with a history of diagnosed subacromial impingement, tendinitis, or adhesive capsulitis were excluded from this study. All participants provided written informed consent; the study protocol was approved by the Pukyong National University Institutional Review Board (No. 1041386-201910-HR-41-01). This study was registered in the Clinical Research Information Service, with registration number KCT0004429.

Based on previous findings demonstrating the effects of the intended scapular posterior tilt on LT muscle activity [10], we calculated that at least 16 participants were required to detect differences in LT muscle activity between PSHA exercises, with and without the intended scapular posterior tilt, at a significance level of *p* < 0.05 with 80% power.

### 2.2. EMG

**Figure 1:**
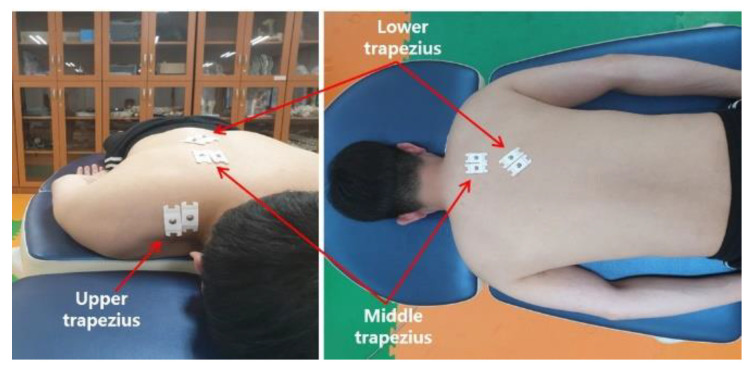
Placements of electrodes for trapezius muscles.

To analyze the EMG activity of the UT, MT, and LT of the dominant shoulder during PSHA exercises, a surface EMG system (wireless miniDTS; Noraxon, Inc., Scottsdale, AZ, USA) was used. Two bipolar surface electrodes were attached at a 2-cm interelectrode distance on the muscle fiber of each muscle, as previously described [24]; the details are provided in Figure 1 and Table 1. EMG data were collected and analyzed using MyoResearch 3 software (Noraxon, Inc., Scottsdale, AZ, USA). EMG data were collected at 1500 Hz, at a bandwidth of 10–450 Hz; they were then full-wave rectified and converted to root mean square values. In a previous study, high or excellent reliability was reported by intraclass correlation coefficient for the measurements of trapezius muscle activation using surface EMG (UT: 0.85–0.97, MT: 0.89-0.97, and LT: 0.86–0.91) [10].

The maximal voluntary isometric contraction (MVIC) value of each muscle was calculated to normalize EMG data during PSHA exercises. Table 1 lists the MVIC methods used for each muscle [25,26]. All participants performed each MVIC trial for 5 s and repeated each trial once; the mean of the two MVIC trials was used to normalize the EMG data for each muscle. EMG activities of the UT, MT, and LT during PSHA exercises were expressed as %MVIC for the data analysis.

### 2.3. Scapular Posterior Tilt Recording

**Figure 2:**
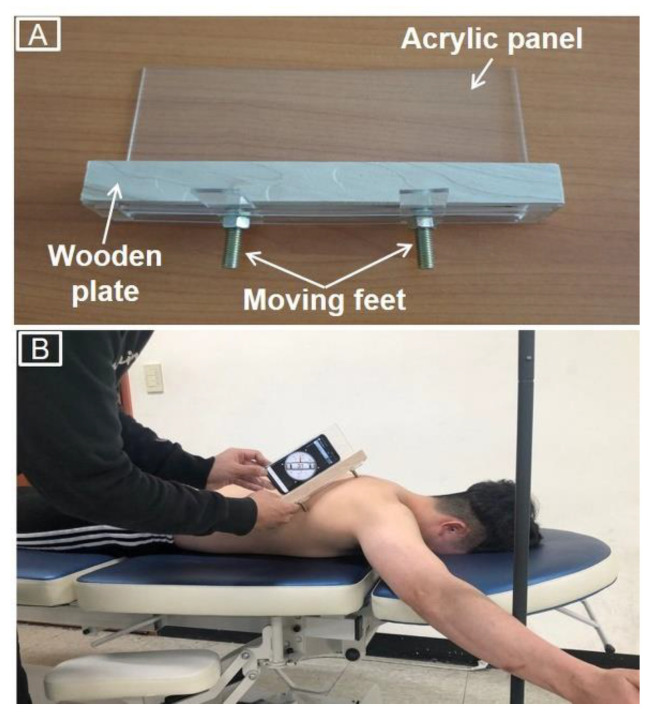
Custom-built measurement plate (**A**) and measurement of scapular posterior tilt using the custom-built measurement plate and an inclinometer (**B**).

Scapular anterior/posterior tilt was measured using a smartphone inclinometer application (Clinometer Level and Slope Finder; Plaincode Software Solutions, Stephanskirchen, Germany) with a custom-built measurement plate [27] consisting of a wooden plate (2.5 cm × 20 cm × 2 cm), acrylic panel (9 cm × 20 cm), and two moving feet (8-mm diameter) (Figure 2A). The wooden plate provided a surface for the smartphone, and the acrylic panel was attached to the plate to maintain vertical alignment of the smartphone; the moving feet were connected to the bottom of the plate and could either move freely or be fixed with nuts for the alignment of the plate with the root of the spine and the inferior angle of the scapula. A previous study used an electromagnetic tracking system and inclinometer with a similar custom-made plate; it showed moderate to good correlation of the scapular anterior and posterior tilt angle measurements during shoulder flexion [27].

To measure scapular posterior tilt, participants assumed the prone position at 125° dominant shoulder abduction while the tested arm was supported on the table. In the condition without the intended scapular posterior tilt, participants maintained preferred scapular position without any the intended scapular motion. In the condition with the intended scapular posterior tilt, following the intended scapular posterior tilt training, participants were in the same position as in the condition without the intended scapular posterior tilt; however, participants were instructed to perform scapular posterior tilt while maintaining the tested arm on the table. During both conditions, an examiner palpated both the root of the spine and the inferior angle of the tested scapula, then placed each of the moving feet of the measurement plate on one of these scapular landmarks. Subsequently, the examiner placed the smartphone on the wooden plate and recorded the angle of scapular posterior tilt using the inclinometer application (Figure 2B). The scapular posterior tilt angle was measured three times each with and without the intended scapular posterior tilt.

### 2.4. The Intended Scapular Posterior Tilt Training

**Figure 3:**
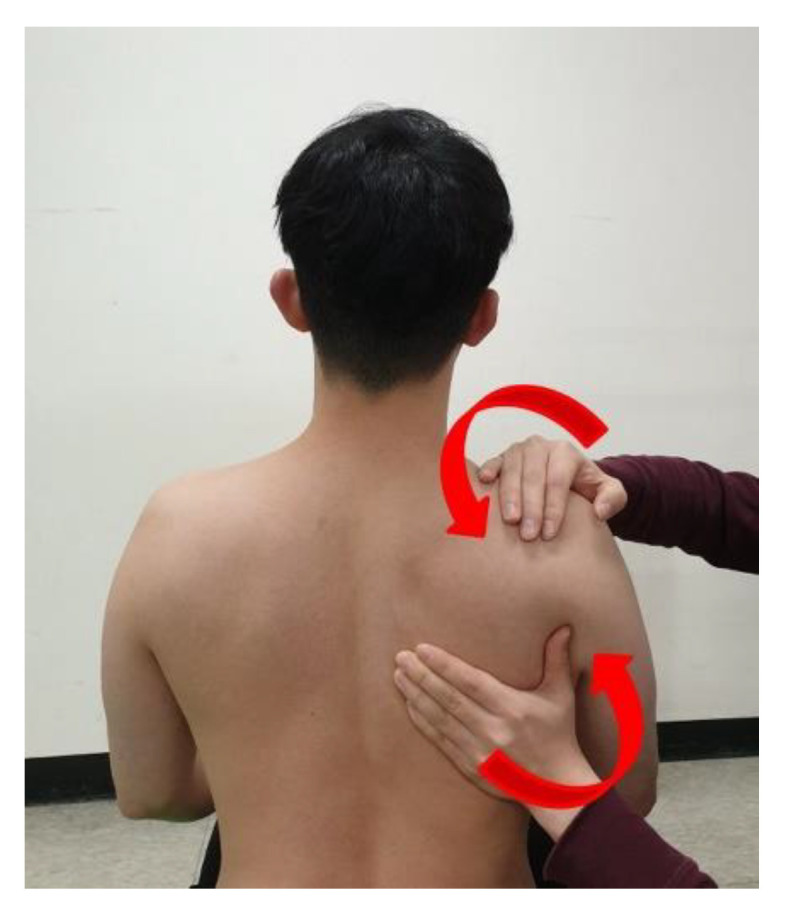
Guidance of scapular posterior tilt movement for the intended scapular posterior tilt training.

During the intended scapular posterior tilt training, participants were asked to pull the coracoid backwards and scapular inferior angle forwards [1,10] in the sitting and prone positions. The correct scapular posterior tilt motion was guided and assisted by an examiner, then performed actively by the participant (Figure 3). Using verbal and tactile cues, trunk extension and/or excessive scapular adduction and depression without posterior tilt were avoided; the correct scapular posterior tilt motion was encouraged [24]. When a participant was able to perform correct scapular posterior tilt and hold this position for 5 s without assistance [24,28], they performed the intended scapular posterior tilt in the prone position for scapular posterior tilt angle measurement; they then performed PSHA combined with the intended scapular posterior tilt, with and without trunk extension. The maximum training period was 10 min.

### 2.5. Prone Shoulder Horizontal Abduction Exercises

**Figure 4:**
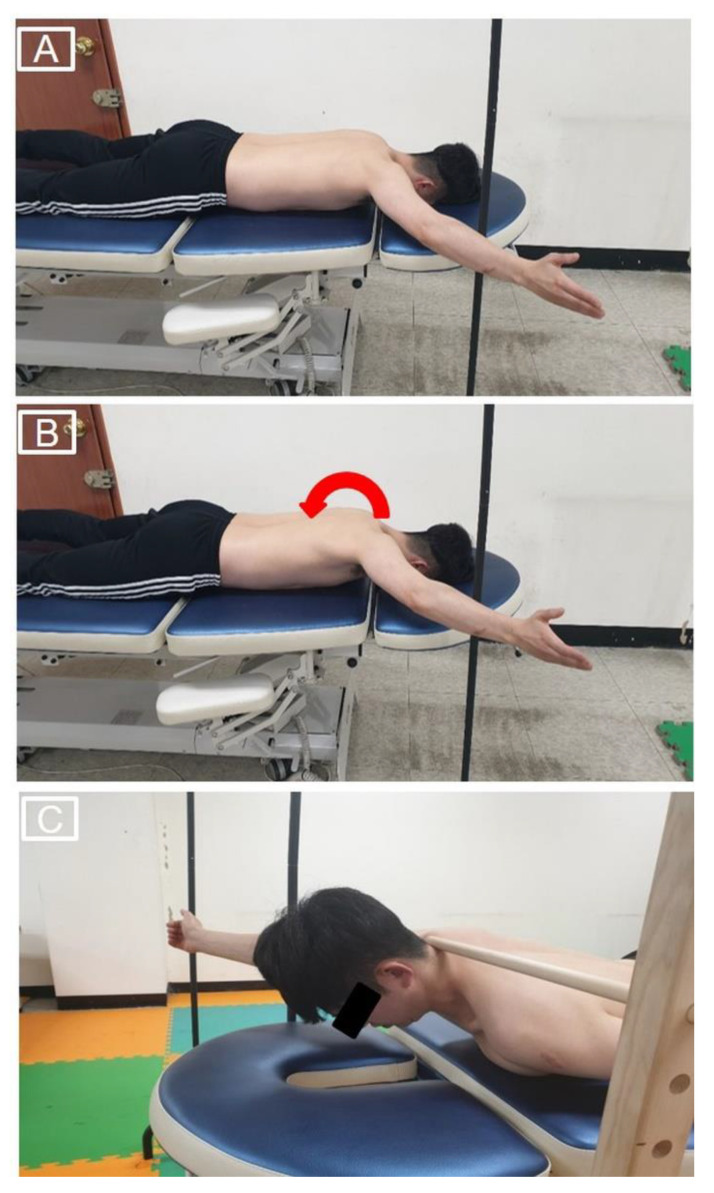
Preferred prone shoulder horizontal abduction (**A**), prone shoulder horizontal abduction with the intended scapular posterior tilt (**B**), and prone shoulder horizontal abduction with the intended scapular posterior tilt and trunk extension (**C**).

Participants performed three PSHA exercises (preferred PSHA, PSHA with the intended scapular posterior tilt, and PSHA with the intended scapular posterior tilt and trunk extension). During all PSHA exercises, a steel pole was used to guide the participant into the 125° shoulder abduction position. For preferred PSHA, the participant lay in the prone position, placed the dominant arm on the table with elbow extension, performed external rotation to 125° shoulder abduction, and then raised the arm as high as possible without trunk rotation (Figure 4A) [15]. For PSHA with the intended scapular posterior tilt, the participant positioned the dominant arm in the same manner as in preferred PSHA, then performed the intended scapular posterior tilt. The participant was instructed to raise the arm while maintaining the intended scapular posterior tilt (Figure 4B). For PSHA with the intended scapular posterior tilt and trunk extension, the participant performed PSHA with the intended scapular posterior tilt as described above, then raised the chest approximately 10 cm from the table [18]. To control the amount of trunk extension, the participant was asked to maintain the 7th cervical vertebra in contact with the target bar during this exercise (Figure 4C). The participant maintained the raised arm position in the end range for 5 s for trapezius muscle EMG activity measurements during each PSHA exercise. Each PSHA exercise was repeated three times. A 1-min rest break was provided between trials and for 5 min between exercises.

### 2.6. Experimental Procedures

**Figure 5:**
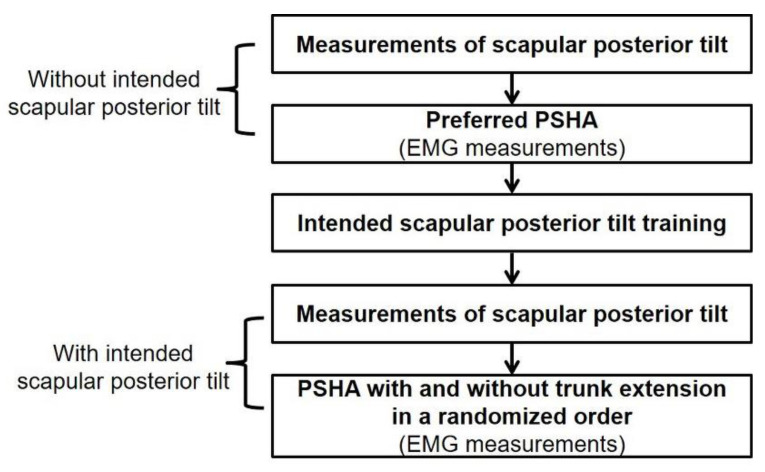
Flow diagram of the experimental procedures. Abbreviations: PSHA, prone shoulder horizontal abduction.

Participants performed preferred PSHA immediately after measurement of the scapular posterior tilt angle in the prone position under the condition without the intended scapular posterior. Next, the intended scapular posterior tilt training was performed to minimize the learning effects; the scapular posterior tilt angle was then measured in the prone position under the condition with the intended scapular posterior tilt. Finally, the participant performed PSHA with and without trunk extension under the intended scapular posterior tilt conditions in randomized order (Figure 5).

### 2.7. Data and Statistical Analyses

For the EMG analysis of UT, MT, and LT muscles, the mean values of three experimental trials of each PSHA exercise (preferred PSHA, PSHA with the intended scapular posterior tilt, or PSHA with the intended scapular posterior tilt and trunk extension) were calculated. For scapular posterior tilt angle analysis, the mean values of three experimental trials of each condition (with or without the intended scapular posterior tilt) were calculated. Normal distributions of outcome variables were verified using the Shapiro–Wilk test; no violations of normality were detected in any of the outcome variables (*p* > 0.05).

Differences in EMG activity among UT, MT, and LT and the UT/MT and UT/LT activity ratios among PSHA exercises were evaluated by one-way repeated analysis of variance with post hoc analysis using the Bonferroni correction. Differences in the scapular posterior tilt with and without the intended scapular posterior tilt were analyzed using paired *t*-tests. PASW software ver. 18.0 (SPSS, Inc., Chicago, IL, USA) was used for all statistical analyses; the significance was evaluated at a level of *p* < 0.05.

## 3. Results

### 3.1. EMG

The trapezius muscle EMG data obtained for all PSHA conditions are shown in Table 2. Significant differences in EMG activity were detected between the UT (*p* < 0.001) and LT (*p* < 0.001) muscles during PSHA exercises. The post hoc analysis revealed less EMG activity in the UT during both preferred PSHA (*p* = 0.005; confidence intervals (CI): −13.23 to −2.23; Effect size (ES): 0.51) and PSHA with the intended scapular posterior tilt (*p* < 0.001; CI: −12.10 to −3.77; ES: 0.51), compared to PSHA with the intended scapular posterior tilt and trunk extension. No significant change in UT muscle activity was detected between the preferred PSHA and PSHA with the intended scapular posterior tilt (*p* = 1.000). Significantly greater LT muscle activity was observed during both PSHA types combined with the intended scapular posterior tilt, with (*p* = 0.010; CI: 2.03 to 16.58; ES: 0.77) and without (*p* < 0.001; CI: 6.41 to 14.70; ES: 0.99) trunk extension, compared to the preferred PSHA. No significant difference in muscle activity was detected between the two PSHA types combined with the intended scapular posterior tilt, with and without trunk extension (*p* = 1.000). There was no significant difference in MT muscle activity among the PSHA exercises (*p* = 0.705).

There were significant differences in the UT/MT ratio (*p* = 0.003) and UT/LT ratio (*p* < 0.001). A post hoc analysis showed a significant reduction in the UT/MT ratio during both preferred PSHA (*p* = 0.017; CI: −0.33 to −0.03; ES: 0.50) and PSHA with the intended scapular posterior tilt (*p* = 0.018; CI: −0.30 to −0.03; ES: 0.46), compared to PSHA with the intended scapular posterior tilt and trunk extension. There was no significant difference in terms of the UT/MT ratio between preferred PSHA and PSHA with the intended scapular posterior tilt (*p* = 1.000). The UT/LT ratio was significantly reduced during PSHA with the intended scapular posterior tilt, compared to both preferred PSHA (*p* = 0.006; CI: −0.12 to −0.02; ES: 0.37) and PSHA with the intended scapular posterior tilt and trunk extension (*p* = 0.001; CI: −0.17 to −0.04; ES: 0.61); however, it did not differ significantly between preferred PSHA and PSHA with the intended scapular posterior tilt and trunk extension (*p* = 0.563).

### 3.2. Scapular Posterior Tilt

The scapular posterior tilt angle was significantly enhanced with the intended scapular posterior tilt, compared to without the intended scapular posterior tilt (17.07 ± 7.29° vs. 14.44 ± 5.56°; *p* = 0.020; CI: 0.47 to 4.79; ES: 0.40).

## 4. Discussion

In this study, we found increased LT muscle activity when the intended scapular posterior tilt was applied during PSHA. However, the additional trunk extension increased the UT muscle activity during PSHA with the intended scapular posterior tilt. This demonstrated that the intended scapular posterior tilt without trunk extension could produce greater selective LT activation.

LT muscle activation was significantly enhanced in the intended scapular posterior tilt with and without trunk extension, compared to preferred PSHA (*p* < 0.05). Previous studies applied the intended neutral scapular position [24] and scapular adduction and depression motion [14] to increase LT muscle activation during PSHA but found that the LT muscle activity did not significantly increase. These studies suggested that a high level of LT muscle activation was already recruited during PSHA with no the intended scapular motion, which produced no significant difference in LT muscle activation between PSHA exercises with and without the intended scapular motion. Our results showed increased LT muscle activation during the intended scapular posterior tilt. Therefore, we presume that the intended scapular motion induced in the previous studies were insufficient to facilitate greater LT muscle activation. In one of these studies [24], participants were instructed to perform selective the intended scapular motion depending on their scapular alignment. Thus, the intended scapular posterior tilt was performed only by participants with scapular anterior tilt alignment during PSHA [24]. Accordingly, LT muscle activation was not thoroughly facilitated in all participants due to inconsistent scapular posterior tilt motion among participants; this likely contributed to the lack of significant difference in LT muscle activity [24]. The other study [14] involved performance of the intended scapular adduction and depression during PSHA to facilitate LT muscle activation. Although these scapular motions are important actions of LT, these motions are also produced by other scapular muscles (e.g., rhomboid, levator scapulae, and latissimus dorsi) [11]. Thus, identifying changes in scapular posterior tilt may be helpful for demonstrating selective LT activation, because LT muscle contraction contributes to scapular posterior tilt [1,2,22,23]. However, the authors of the prior study did not measure changes in scapular posterior tilt caused by the intended scapular motion, whereas the present study detected the significant enhancement of the scapular posterior tilt angle during the intended scapular posterior tilt in the prone position (*p* = 0.020). Considering the influence of the intended scapular posterior tilt on scapular kinematics in the present study, the intended scapular posterior tilt motion appears to effectively facilitate greater LT muscle activation during PSHA.

In the present study, no significant change in LT muscle activation was found between the intended scapular posterior tilt with and without trunk extension (*p* = 1.000). LT acts on various scapular motions and scapular stabilization; it also facilitates thoracic extension [26]. Therefore, trunk extension facilitates LT activation [18,19]. However, we detected no significant enhancement of LT muscle activity in the intended scapular posterior tilt with trunk extension, compared to the intended scapular posterior tilt without trunk extension during PSHA. In the present study, participants were instructed to perform the intended scapular posterior tilt, followed by trunk extension. Given that we observed similar LT muscle activity between the intended scapular posterior tilt with and without trunk extension, we presume that volitional preemptive LT contraction (due to the intended scapular posterior tilt motion) met the LT muscle activation required for trunk extension, which influenced our findings.

We observed significant enhancements of UT/LT (*p* = 0.001) and UT/MT (*p* = 0.018) ratios in the intended scapular posterior tilt with trunk extension compared to the intended scapular posterior tilt without trunk extension; no significant difference in LT or MT EMG activity was detected between these two conditions (*p* = 1.000). These findings may have been caused by enhanced UT muscle activity during trunk extension (*p* < 0.05). The UT muscle originates at the external occipital protuberance, ligamentum nuchae, and superior nuchal line; thus, it is the only trapezius muscle involved in head and cervical movements, including cervical extension [11,26]. During the intended scapular posterior tilt with trunk extension, the participant was encouraged to hold the head and neck without support; thus, the participant recruited UT muscle activation to hold the head and neck position against gravity during trunk extension [18], which led to greater UT/LT and UT/MT ratios, as well as greater UT muscle activity during trunk extension.

Increased UT/LT and UT/MT ratios are critical features in individuals with shoulder injuries [6,8]. During normal scapulothoracic motion, arm elevation is accompanied by scapular upward rotation, external rotation, and posterior tilt [4,6,22,29]. However, higher UT muscle activity produces excessive scapular anterior tilt with clavicular elevation, consequently leading to scapular dyskinesia, which can be related to shoulder injuries [6,29]. Thus, exercises inducing the reduction of UT muscle activity and enhancements of LT and MT muscle activity are emphasized in clinics. However, MT muscle activity was not significantly enhanced when the intended scapular posterior tilt was applied during PSHA in the present study; this is presumably because scapular adduction, the primary action of the MT, is not emphasized during the intended scapular posterior tilt. In contrast to the MT, we observed significantly greater LT muscle activity, together with a significantly lower UT/LT ratio, during the intended scapular posterior tilt without trunk extension. Based on these findings, PSHA combined with the intended scapular posterior tilt should be recommended for selective strengthening of the LT.

Several limitations of the present research should be addressed in future researches. First, muscle activation of other periscapular muscles were not measured during the exercises. Second, the scapular posterior tilt angle could not be measured during dynamic trunk extension. Since the scapular posterior tilt angle was calculated as the angle of scapular inclination with respect to the horizontal plane, the calculated scapular tilt angle was influenced by both scapular motion and trunk extension. Therefore, future research should apply a three-dimensional motion analysis system to measure the scapular posterior tilt angle during exercises. Third, no external load was added during exercises in this research. Since the response of the muscles may vary depending on the additional external load, it is necessary to consider the external load in future research. Moreover, future research comparing the dominant side and the nondominant side or applying various feedback strategies for scapular posterior tilt would also be valuable. Finally, future research should identify the effects of the intended scapular posterior tilt during other LT strengthening exercises, as well as on symptomatic subjects.

## 5. Conclusions

Our findings indicate that the intended scapular posterior tilt motion without trunk extension enhances the LT muscle activity while reducing the UT/LT ratio during PSHA. These results suggest that the intended scapular posterior tilt motion may be a useful strategy to selectively enhance LT muscle activation during exercise.

## Figures and Tables

**Table 1:** Placements of electrodes and maximal voluntary isometric contraction methods for each muscle.

Muscles	Placements of Electrodes	MVIC Methods
UT	Midway between 7th cervical vertebrae and acromion along the line of muscle fiber	Participant performed shoulder elevation, ipsilateral cervical side-flexion, and contralateral cervical rotation against resistance applied to the shoulder and head by an examiner in the opposite direction of movements of participant in the sitting
MT	Midway between 3rd thoracic vertebrae and root of scapular spine along the line of muscle fiber	Participant performed shoulder horizontal abduction in prone with 90° of shoulder abduction and 90° of elbow flexion against resistance applied to the distal humerus by an examiner
LT	Midway between 7th thoracic vertebrae and intersection of the spine and medial boarder of the scapula along the line of muscle fiber	Participant performed PSHA with shoulder external rotation and elbow extension while diagonally placing arm overhead in line with LT muscle fiber against resistance applied to the forearm by an examiner

LT: lower trapezius; MT: middle trapezius; MVIC: maximal voluntary isometric contraction; PSHA: prone shoulder horizontal abduction; UT: upper trapezius.

**Table 2:** Muscle activity during exercises.

Measure	Preferred PSHA	PSHA with The Intended Scapular Posterior Tilt	PSHA with The Intended Scapular Posterior Tilt and Trunk Extension	*p*
UT (%MVIC)	28.82 ± 12.82 ^3^	28.61 ± 14.07 ^3^	36.55 ± 16.87 ^2^	0.001 ^1^
MT (%MVIC)	55.20 ± 15.17	54.16 ± 16.53	56.68 ± 20.96	0.710
LT (%MVIC)	65.67 ± 11.18 ^3^	76.23 ± 10.13 ^2^	74.98 ± 12.89 ^2^	<0.001 ^1^
UT/MT activity (ratio)	0.54 ± 0.26 ^3^	0.55 ± 0.27 ^3^	0.72 ± 0.42 ^2^	0.021 ^1^
UT/LT activity (ratio)	0.45 ± 0.20	0.38 ± 0.17 ^2,3^	0.49 ± 0.19	<0.001 ^1^

^1^ Significant differences among exercises, *p* < 0.05. ^2^ Significant differences from preferred PSHA, *p* < 0.05. ^3^ Significant differences from PSHA with the intended scapular posterior tilt and trunk extension, *p* < 0.05. LT: lower trapezius; MT: middle trapezius; MVIC: maximal voluntary isometric contraction; PSHA: prone shoulder horizontal abduction; UT: upper trapezius.

## Data Availability

Not applicable.
